# Larvicidal activity of essential oils and nanoemulsions against *Culex pipiens* larvae (Diptera: Culicidae)

**DOI:** 10.1038/s41598-026-54655-1

**Published:** 2026-06-04

**Authors:** Hoda S. M. Abdel-Ghany, Fathalla Ayoob, Baiome Abdelmaguid Baiome, Shaimaa A. Allam, Abdelghany A. Youssef, Mohamed M. Kabadaia, Sobhy Abdel-Shafy

**Affiliations:** 1 Department of Parasitology and Animal Diseases, Veterinary Research Institute, National Research Centre, 33 El Bohouth St., Dokki, Giza, Egypt; 2 Ticks and Tick-Born Diseases Research Unit, Veterinary Research Institute, National Research Centre, 33 El Buhouth St., Dokki, Giza, Egypt; 3 Chemistry of Tanning Materials and Leather Technology Department, Chemical Industries Research Institute, National Research Centre, 33 El Bohouth St., Dokki, Giza, Egypt; 4 Department of Molecular Biology, Genetic Engineering and Biotechnology Research Institute, National Research Centre, Dokki, Giza, Egypt; 5 Medicinal and Aromatic Plants Research Department, Pharmaceutical Industries Research Institute, National Research Centre, Dokki, Giza, Egypt; 6 Zoology and Entomology Department, Faculty of Science, Al-Azhar University, Nasr City, Cairo 11884 Egypt

**Keywords:** Essential oils, *Culex pipiens*, Insecticide, Glutathione-*S*-transferase (GST), Nanoemulsion, Biochemistry, Biological techniques, Biotechnology, Environmental sciences, Plant sciences, Zoology

## Abstract

Mosquitoes and mosquito-borne diseases are a growing global challenge. Vector control strategies have recently transitioned from chemical insecticides to botanical products. This study evaluated the larvicidal efficacy of dill, lime, and wormwood essential oils (EOs), along with their combinations and nanoemulsions (NEs), against *Culex pipiens* third-instar larvae over 48 h. Gas chromatography mass spectrometry (GC–MS) analysis revealed major components for dill EO (Apiol 25.46%, Carvone 24.13%, and D-Limonene 23.47%), lime EO (D-Limonene 24.16%, α-Terpineol 13.12%, and β-Pinene 11.98%), and wormwood EO (davanone 33.8%, and camphor 25.67%). The TEM image showed spherical NE droplets (40–160 nm). The droplet size and polydispersity index (PDI) were (164 nm and 0.2), (211.6 nm and 0.2), and (160.8 nm and 0.6) for dill, lime, and wormwood NEs, respectively. Based on LC_50_ values after 30 min, the dill EO showed the highest larvicidal activity (LC_50_ = 286.9 ppm; confidence interval (CI) 226.85-341.52 ppm), followed by dill NE (LC_50_ = 518.9 ppm; CI 290.4 -765.1 ppm) and then (dill + lime) binary combination (LC_50_ = 1502 ppm; CI 1403.9-1605.4 ppm). The EO showed faster larvicidal activity than NEs, particularly within 30 min. NEs didn’t significantly enhance efficacy over crude EOs. Enzyme assays showed variable effects: dill, lime, and wormwood EOs reduced GST activity, whereas their corresponding NEs generally increased enzyme activity at most exposure times. These findings indicate that botanical formulations, particularly those derived from dill, may serve as promising biodegradable alternatives for vector control. However, optimizing essential oil ratios is needed to maximize synergistic effects, and comprehensive assessments of toxicity and environmental impact are required before large-scale application.

## Introduction

Mosquitoes are considered among the most hazardous insect pests due to their ability to transmit serious diseases such as malaria, dengue, and yellow fever, posing major health risks to both humans and animals^1,2^. They serve as vectors for numerous pathogens, including viruses, bacteria, and parasites, resulting in significant public health and economic burdens in endemic regions. *Culex* mosquitoes are widely distributed in tropical and subtropical areas and commonly breed in contaminated environments, including clogged drains and poorly maintained septic tanks^3^.

Chemical insecticides, particularly organophosphates and pyrethroids, remain the primary method for mosquito control worldwide, including Egypt. Organophosphates are commonly applied outdoor environments to target mosquito larvae, whereas pyrethroids are primarily used indoors to kill adult mosquitoes^4^.

However, overusing these insecticides leads to resistance and harms the environment and non-target species^5,6^. Therefore, it is essential to explore alternatives that significantly impact mosquitoes but are eco-friendly^7^.

Botanical insecticides, particularly essential oils, are considered biodegradable and generally safer for humans and the environment than conventional chemical insecticides. Moreover, their multiple modes of action may slow the development of insecticide resistance^8^. Due to their lipophilic nature, plant essential oils can readily penetrate cell membranes and interfere with various biochemical, physiological, and behavioral processes in insects^9^.

Dill (*Anethum graveolens* L.) is a member of the Apiaceae family. It is an annual herb, native to Middle East, including Egypt, and has been valued for its medicinal properties^10^. It has sedative effects and is used to treat intestinal spasms, colic pain, and liver diseases^11^. Dill seed extracts possess antibacterial, antispasmodic, antioxidant, and antimicrobial activities^12^, as well as insecticidal properties^10,13,14^. Additionally, lime (*Citrus aurantifolia*) belongs to the Rutaceae family. It has potent insecticidal and antibacterial properties^15,16^ that make it useful for natural disease and pest management. Also, it has larvicidal and ovicidal effects against mosquitoes such as *Aedes aegypti*^17^. Furthermore, wormwood (*Artemisia absinthium*) is a medicinal plant in the Asteraceae family. Its leaf powder has been used to treat intestinal worms and gastrointestinal issues^18,19^. It exhibits potent larvicidal effects against mosquito larvae^20,21^. These oils contain diverse blends of monoterpenes, phenols, and sesquiterpenes, and exhibit a range of bioactive properties. They show insecticidal, repellent, and antifeedant activity, and can disrupt insect growth, reduce adult fertility, and inhibit oviposition^22,23^.

Previous studies have shown that several essential oils act on multiple target sites in insects, including the nervous and digestive systems, developmental processes, oviposition behavior, ion channels, and specific enzymes^24,25^. Another promising approach in mosquito control is combining essential oils. This can produce synergistic effects, as reported by Wangrawa et al.^26^, Mahran et al.^27^, and Vivekanandhan et al.^28^, or antagonistic interactions, as observed by Andrade-Ochoa et al.^29^, Sarma et al.^17^, and Mahanta and Khanikor^30^.

In this study, the selection of dill, lime, and wormwood was based on their previous insecticidal properties, attributed to high concentrations of bioactive terpenoids, coupled with their widespread availability.

Despite the promise of EOs as biopesticides, their practical use remains limited by high volatility, low water solubility, and rapid degradation under environmental conditions^31^. Although many studies emphasize improved efficacy as the main advantage of nanoformulations, their key rationale lies in overcoming the inherent limitations of crude EOs: high volatility, low water solubility, and rapid degradation under environmental conditions^32,33^. This study used NEs to overcome these physicochemical limitations. Reducing EO droplets to the nanoscale improves bioavailability and enhances penetration through the larval cuticle, which is essential for mosquito control^33–35^.

Recent studies have demonstrated the larvicidal potential of several essential oil nanoemulsions, including those formulated from orange, *Cymbopogon commutatus*, and clove oil^36–38^. Furthermore, nanoemulsions of *Cinnamomum zeylanicum* evaluated against *Anopheles stephensi* larvae^39^, while *Murraya koenigii* NE exhibited activity against *Aedes aegypti*^40,41^. Similarly, *Lantana camara* NE showed activity against *Anopheles culicifacies* larvae^42^.

To address the limitations of essential oils, specifically their high volatility and poor water solubility, and the lack of comparative data regarding the impact of these EOs on the enzymatic activity of *C. pipiens* larvae, this study aims to characterize the chemical profiles of three EOs, develop nano-formulations, and evaluate their effects on both mortality and enzymatic activity (GST) in *Culex pipiens* larvae.

## Materials and methods

### Plant materials

Seeds of *Anethum graveolens* L. (dill) were obtained from the Department of Medicinal and Aromatic Plants, Ministry of Agriculture, Giza, Egypt. Peels of *Citrus aurantifolia* (lime) fruits were collected from the farm of the National Research Centre (NRC) in Nubaria, Egypt, an arid region located approximately 130 km west of Cairo. Fresh aerial parts of *Artemisia absinthium* (wormwood) were collected during the flowering stage from the foothills of the Faifa Mountains, south of Jazan City, Saudi Arabia. Dill and lime were identified by Dr. Mona Mohamed Marzouk, a professor of Phytochemistry and Plant Systematics at the National Research Centre (NRC). The voucher specimens (M293 for dill and M294 for lime) were deposited in the herbarium of NRC (CAIRC). Wormwood was identified by Dr. M. Remesh, Department of Biology, Faculty of Science, Jazan University, Saudi Arabia. A voucher specimen (No. 117) was deposited in Jazan University’s Herbarium (JAZUH).

### Essential oil hydro-distillation

Essential oils were extracted from air-dried dill seeds, fresh lime peels, and air-dried aerial parts of wormwood by hydro-distillation using a Clevenger-type apparatus. Briefly, 250 g of each plant material was distilled in triplicate for 3 h, or until no further oil was recovered^43^. The obtained oils were dried over anhydrous sodium sulfate and appeared as yellow (dill), greenish yellow (lime), and deep blue (wormwood) oils. All EO samples were stored in dark brown glass vials at 4 °C until further analysis.

### Gas chromatography mass spectrometry (GC–MS) analysis

The samples were analyzed using an Agilent 8890 GC System gas chromatograph coupled to an Agilent 5977B GC/MSD mass spectrometer, equipped with a HP-5MS fused silica capillary column (30 m × 0.25 mm i.d. × 0.25 μm film thickness). The GC oven was initially set to 50 °C, then ramped up to 220 °C at 5 °C/min, followed by a ramp to 280 °C at 15 °C/min, where it was held for 7 min. Helium carrier gas flowed at 1.1 mL/min. The essential oil was diluted in diethyl ether (30 µL/mL), and 1 µL was injected (split ratio 1:50) at 230 °C. Electron impact (EI) mass spectra were obtained at 70 eV, scanning m/z 39–500. The sample was manually injected in splitless mode and analyzed by flame ionization detection (FID) using HP ChemStation software on an HP 5980 GC, employing the same column type and temperature program as used for GC/MS.

### Identification of the essential oil components

Essential oil components were identified by comparing their mass spectra to those in a computer library or to authentic compounds. Then, their retention indices were matched with authentic compounds, literature data, or compared to literature mass spectra^44^. Components were identified by comparing retention times with standards and matching mass spectral data with MS libraries (NIST/NBS and Wiley 275.l), aided by computer search and literature review^44^. Area percentages from FID were used for quantitative analysis. Retention indices (RI) were determined by injecting a homologous series of n-alkanes, C8-C20 and compared to published values to confirm identification^44^. Computer matching was done using the Wiley GC/MS Library and the Mass Finder 3 Library.

### Nanoemulsion preparation

The essential oil was mixed with the nonionic surfactant Tween 80, a sorbitan ester derivative, using a low-energy emulsification method to produce a homogeneous nanoemulsion with a small droplet size. Tween 80 was selected due to its high hydrophilic–lipophilic balance (HLB) value (15), which promotes the formation of stable oil-in-water (O/W) nanoemulsions and has demonstrated biocompatibility in food and pharmaceutical applications. Spontaneous emulsification was performed by preparing separate organic phases for dill, lime, and wormwood EOs. Tween 80 was mixed with each EO at a 1:1 (w/w) ratio, while the EO concentration was maintained at 20% in all formulations. The final nanoemulsion composition consisted of 20% essential oil, 20% surfactant, and 60% water (w/w).

The organic phase mixture was added dropwise to distilled water (aqueous phase) at a rate of 1 mL/5 min under continuous stirring using a high-speed stirrer at 2000 rpm and room temperature. Following this process, NEs were successfully formed for each EO. The prepared NEs were then continuously stirred on a magnetic stirrer for 24 h at room temperature to obtain stabilized functionalized NEs^45,46^.

### Characterization of essential oil nanoemulsions

#### Transmission electron microscope (TEM)

Transmission electron microscopy images were obtained using a JEOL JEM-2100 TEM. For analysis, diluted NE samples were deposited onto carbon-coated copper grids and allowed to air-dry at room temperature for 10 min before imaging^47^.

#### Droplet size analysis

Particle size distribution was determined using a Malvern Zetasizer 3000 HAS via dynamic light scattering (DLS) at 23 °C, with a 2-minute run time, water as solvent, and 1 mg/mL sample concentration^48,49^.

### Mosquito

*Culex pipiens* 3rd instar larvae were obtained from the Mosquito Research Department, Research Institute of Medical Entomology, Al Agouzah, Giza Governorate, Egypt. A pilot study was carried out to determine the range of concentrations used in the bioassay.

### Larvicidal bioassay

The larvicidal activity of essential oils (either individual or in binary combination (ratio 1:1) and their nanoemulsions against *C. pipiens* third instar larvae was demonstrated according to WHO standard guidelines^50^. A total of 2400 larvae were used in the bioassay experiments. Five concentrations of each tested material (4000, 2000, 1000, 500, 250 ppm) were used. Each concentration was tested in 5 independent replicates with 10 larvae / replicate. Essential oils and their combinations were formulated using 1% Tween 80 as an emulsifier and distilled water for NE concentrations. Two control groups were used: 1% Tween 80 for essential oils (EOs) and their combinations, and distilled water for nanoemulsions (NEs). The positive control (Deltamethrin 5%) was used at a concentration of 6.5 ppm. All experiments were conducted under controlled environmental conditions (27 ± 2 °C). The bioassay was performed using plastic cups containing 50 ml of the tested materials. Larval mortality was recorded at 0.5, 1, 3, 6, 24, and 48 h post-exposure.

### Determination of enzymatic activity

#### Preparation of mosquito homogenates

A total of 1200 *C. pipiens* third-instar larvae were used (50 larvae per treatment per time) to evaluate GST activity. The treatments included three EOs, three NEs (each at LC_50_), and two controls (1% Tween 80 and distilled water), with measurements taken at 0, 30, and 60 min. Mosquito larvae (weighing 0.1–0.15 g) were homogenized in 50 mM potassium phosphate buffer (pH 7.5) to estimate total protein and glutathione S-transferase (GST) activity. The homogenates were centrifuged at 11,000 ×g for 15 min. The supernatants were filtered through glass wool to remove lipids, yielding cytosolic fractions (crude homogenates), which were stored at -20 °C for further analysis^51^.

#### Glutathione *S*-transferase measurement

The glutathione *S*-transferase (GST) activity was measured spectrophotometrically using 1-chloro-2,4-dinitrobenzene (CDNB) as the substrate and reduced glutathione (GSH). Thioether formation was monitored by tracking the change in absorbance at 340 nm at 25 °C, as described by Habig et al.^52^. The assay mixture (1 mL total volume) contained 100 mM potassium phosphate buffer (pH 6.5), 1 mM CDNB (in ethanol, < 4% final concentration), 1 mM GSH, and enzyme solution. Absorbance increase at 340 nm was monitored against a control (buffer instead of enzyme). The product’s molar extinction coefficient was 9.6 mM⁻¹ cm⁻¹. One GST activity unit is defined as the enzyme amount catalyzing 1 µmol thioether formation per minute.

#### Protein determination

Protein concentration in mosquito larvae homogenates was determined using the Bradford^53^ method, with BSA as a standard. A series of BSA standard solutions was used for calibration. BSA (10%) was dissolved in 10 mL of distilled water, and then serial dilutions with water were prepared for standard protein calibration covering the concentrations from 1 to 10 µg ml − 1 of BSA. One hundred milligrams of Coomassie Brilliant Blue (CBB, G-250) was dissolved in 50 mL of 95% ethanol. To this solution, 100 mL of orthophosphoric acid (85% w/v) was added, and the final volume was adjusted to 1 L with distilled water. The dye solution was filtered and kept for two weeks at 25 ℃ before use. Then, a known volume of the protein sample was adjusted to a total volume of 1 ml with distilled water. One ml of the dye solution was added to the sample and mixed. The absorbance of the blue color formed was measured at 595 nm within 1 h against a blank prepared by mixing 1 ml of distilled water with 1 ml of the dye solution. A standard protein covering the range of 1 to 10 µg in 1 ml using BSA was carried out.

### Statistical analysis

Mortality percentage and enzyme activity of 3rd-instar larvae were statistically analyzed using one-way ANOVA, followed by Tukey’s tests (SPSS version 20). The effects of concentration, exposure time, and essential oil formulation on the mortality of 3rd instar larvae were analyzed using three-way ANOVA. Effects of exposure time and essential oil formulation on GST activity in 3rd instar larvae were analyzed using two-way ANOVA. Mortality data were subjected to Abbott’s correction and Probit analysis using Ldp Line^®^ software to estimate the median lethal concentrations (LC_50_ value) along with their confidence intervals (CI) and Slop^54^. To evaluate the synergistic or antagonistic activity of essential oil combinations, the synergistic factor (SF) was calculated as follows: SF = LC_50_ of individual oil / LC_50_ of combination. SF› 1  synergistic; SF‹1 indicates antagonistic.

## Results

### GC-mass analysis

Dill EO yield was 2.39% (v/w), with 14 components identified (Table 1). Four major compounds, Apiol (25.46%), Carvone (24.13%), D-limonene (23.47%), and trans-dihydrocarvone (17.05%) constituted 90.11% of the oil. Cis-Dihydrocarvone accounted for 5.12%, and nine minor components comprised the remaining 4.77%. GC-MS analysis of lime EO identified 23 components comprising 100% of the oil (Table 2), with a yield of 0.47% (v/w). The five major constituents were D-limonene (24.16%), α-terpineol (13.12%), β-pinene (11.98%), γ-terpinene (8.44%), and terpinen-4-ol (6.92%), together accounting for 64.62% of the total. Wormwood EO yield was 0.78% (v/w). GC-MS analysis identified davanone (33.8%), camphor (25.67%), and chamazulene (7.47%) as major constituents. Minor components included linalool (4.58%), terpinen-4-ol (4.41%), bornyl acetate (3.78%), ethyl cinnamate (3.04%), camphene (2.74%), α-pinene (2.41%), (E)-nerolidol (2.31%), and cis-sabinene hydrate (2.18%) (Table 3).

**Table 1:** Chemical composition of dill essential oil.

Peak	RT	RI_Cal_.	RI_Lit_.	Oil constituent	EO components (%)
1	7.685	990	991	β-Myrcene	0.26
2	8.066	1005	1005	α-Phellandrene	0.85
3	8.794	1032	1028	D-Limonene	23.47
4	10.382	1090	1087	p-cymenene	0.35
5	11.624	1135	1134	E)-Limonene oxide	0.23
6	13.455	1200	1193	cis-Dihydrocarvone	5.62
7	13.732	1211	1202	trans -Dihydrocarvone	17.05
8	13.957	1219	1214	Dihydrocarveol	0.5
9	14.084	1223	1217	trans-Carveol	0.38
10	14.362	1234	1227	cis-Carveol	1.03
11	14.858	1252	1241	Carvone	24.13
12	21.881	1526	1519	Myristicin	0.51
13	22.661	1559	1558	Elemicin	0.18
14	24.422	1635	1624	Dill Apiol	25.46

RT: retention time, RI_Cal_: retention index calculated using *n*-alkanes series (8:20), RI_Lit_ : retention index from literature, EO: essential oil.

**Table 2:** Chemical composition of lime essential oil.

Peak	RT	RI_Cal_.	RI_Lit_.	Oil constituent	EO components (%)
1	6.305	933	932	α-Pinene	2.3
2	7.275	973	969	Sabinene	1.69
3	7.379	977	974	β-Pinene	11.98
4	7.68	990	991	β-Myrcene	1.09
5	8.378	1016	1018	α-Terpinene	1.38
6	8.605	1025	1026	p-Cymene	1.51
7	8.76	1031	1031	D-Limonene	24.16
8	9.21	1047	1040	β-Ocimene	0.46
9	9.539	1059	1059	γ-Terpinene	8.44
10	10.346	1089	1088	α Terpinolene	2.43
11	10.66	1101	1099	Linalool	1.7
12	12.581	1179	1166	Borneol	1.15
13	12.895	1181	1177	Terpinen-4-ol	6.92
14	13.293	1195	1189	α-Terpineol	13.12
15	14.267	1230	1228	Nerol	1.65
16	14.622	1243	1241	Neral	3.21
17	14.981	1256	1255	Geraniol	2
18	15.424	1281	1270	Geranial	4.42
19	19.398	1424	1418	β-Caryophyllene	1.55
20	19.744	1438	1434	trans-α-Bergamotene	2.05
21	21.494	1509	1504	α-Farnesene	2.08
22	21.54	1511	1509	β-Bisabolene	3.64
23	22.782	1564	1560	Germacrene B	1.07

RT: retention time, RI_Cal_: retention index calculated using *n*-alkanes series (8:20), RI_Lit_ : retention index from literature, EO: essential oil.

**Table 3:** Chemical composition of wormwood essential oil.

No	RT	RI_Cal_.	RI_Lit_.	Oil constituent	EO components (%)
1	6.34	934	934	α-Pinene	2.41
2	6.588	944	946	n-Propyl 2-methylbutyrate	0.84
3	6.703	949	950	Camphene	2.74
4	7.708	991	991	β-Myrcene	1.14
5	8.407	1018	1017	α-Terpinene	0.7
6	8.627	1026	1024	p-Cymene	1.22
7	8.731	1030	1030	D-Limonene	0.59
8	9.551	1060	1060	γ-Terpinene	1.42
9	9.811	1069	1066	cis-Sabinene hydrate	2.18
10	10.683	1110	1099	Linalool	4.58
11	11.994	1148	1144	(+)-Camphor	25.67
12	12.589	1169	1166	Borneol	0.9
13	12.9	1181	1177	Terpinen-4-ol	4.41
14	15.875	1289	1285	Bornyl acetate	3.78
15	18.26	1380	1374	Ethyl (Z)-cinnamate	0.8
16	20.512	1469	1464	Ethyl cinnamate	3.04
17	22.857	1567	1564	(E)-Nerolidol	2.31
18	23.446	1592	1588	Davanone	33.8
19	26.732	1740	1727	Chamazulene	7.47

RT: retention time, RI_Cal_: retention index calculated using *n*-alkanes series (8:20), RI_Lit_ : retention index from literature, EO: essential oil.

The chemical classes of dill, lime, and wormwood EOs are summarized in Table 4. Terpenes dominated dill EO composition, accounting for 73.8% of total volatiles. Oxygenated monoterpenes (OM) comprised 48.9% and monoterpene hydrocarbons (MH) comprised 24.9%. Phenylpropanoids were also substantial at 26.2%. Lime EO was composed exclusively of terpenes. Monoterpene hydrocarbons accounted for 55.4%, followed by oxygenated monoterpenes (34.2%) and sesquiterpenes (10.4%), totaling 100%. Wormwood EO was composed of 95.3% terpenes, including oxygenated monoterpenes (41.5%), oxygenated sesquiterpenes (36.1%), monoterpene hydrocarbons (10.2%), and sesquiterpene hydrocarbons (7.5%; primarily chamazulene). Phenylpropanoids accounted for 3.8%, and fatty acid derivatives, including n-propyl 2-methylbutyrate (0.84%), comprised 0.84% (Table 4).

**Table 4:** Chemical classes of dill, lime, and wormwood essential oils.

Chemical class	Dill	Lime	Wormwood
Monoterpene hydrocarbon (MH)	24.9	55.4	10.2
Oxygenated monoterpenes (OM)	48.9	34.2	41.5
Sesquiterpene hydrocarbons	0.00	10.4	7.5
Oxygenated sesquiterpene	0.00	0.00	36.1
phenylpropanoids	26.2	0.00	3.8
Others	0.00	0.00	0.8
Total	100	100	100

### Characterization of nanoemulsion

#### TEM analysis of essential oil nanoemulsions

**Fig. 1:**
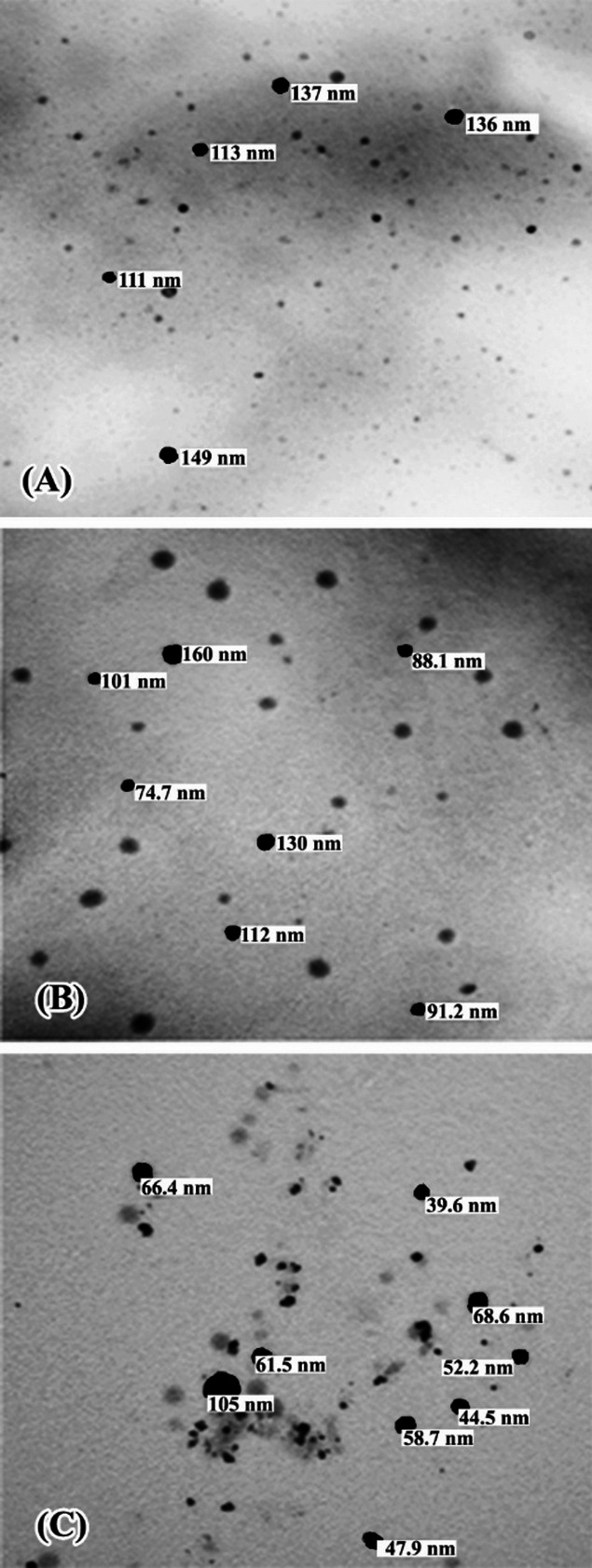
TEM images of essential oil nanoemulsions: (**a**) dill, (**b**) lime, (**c**) wormwood. NEs are spherical, 40–160 nm in size.

TEM analysis revealed that the NEs were spherical with particle sizes ranging from 40 to 160 nm (Fig. 1).

#### Droplet size distribution analysis

**Fig. 2:**
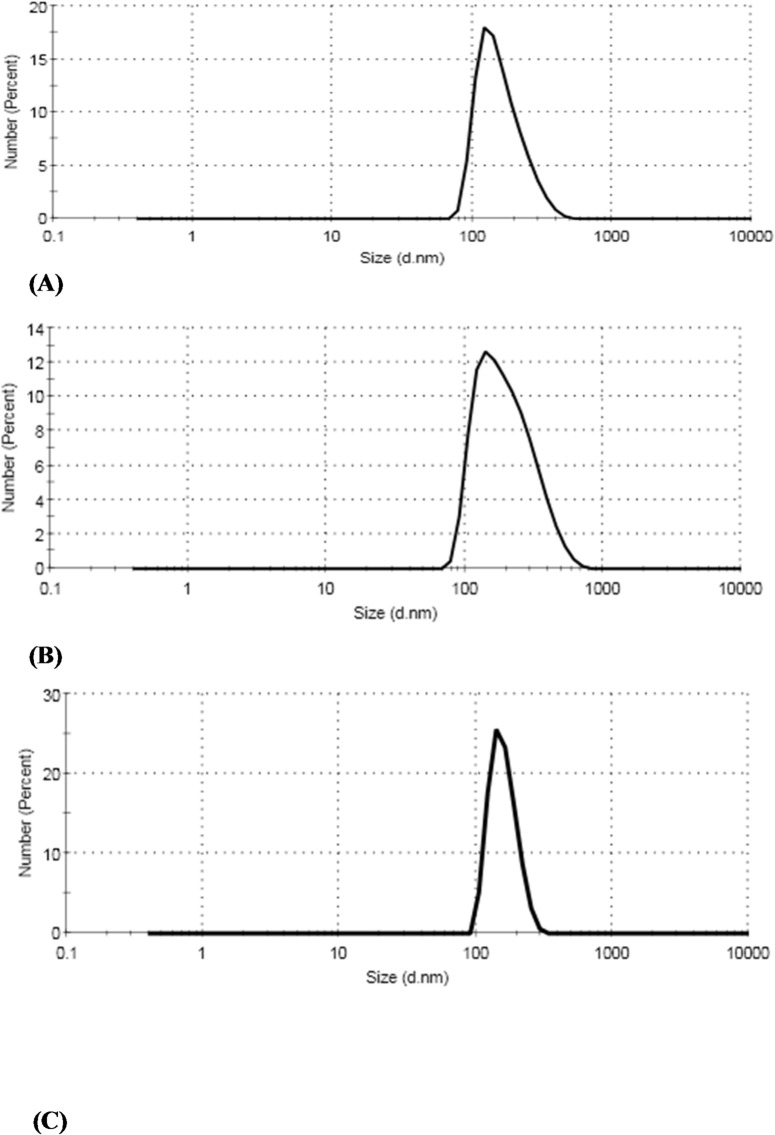
Particle size analysis of essential oil nanoemulsions: (**a**) dill, (**b**) lime, (**c**) wormwood.

**Fig. 3:**
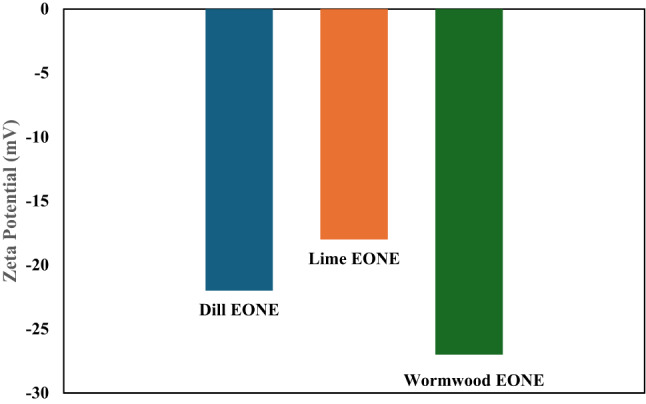
Zeta potential of dill, lime, and wormwood essential oil nanoemulsions; EONE essential oil nanoemulsion.

The size distribution of the NE droplets was estimated. The mean droplet diameters were 164.2 ± 62.97 nm, 211.6 ± 102.8 nm, and 160.8 ± 0.038 nm for dill, lime, and wormwood, respectively (Table 5; Fig. 2). The PDI value ranges from 0.2 to 0.6, indicating system stability due to its lower values. The zeta potential values proved the high stability of the prepared formulations and surface charge characteristics. The dill, lime, and wormwood nanoemulsion formulations exhibited a negative zeta potential of − 22 ± 2.24 mV, − 18 ± 3.14 mV, and − 7 ± 3.16 mV, respectively (Table 5; Fig. 3).

**Table 5:** Droplet size, polydispersity index, and zeta potential of dill, lime, and wormwood nanoemulsion.

Sample	Particle size mean (nm)	Polydispersity index (PDI)	Zeta potential (mV)
Dill nanoemulsion	164 ± 62.97	0.237	−22 ± 2.24
Lime nanoemulsion	211.6 ± 102.8	0.221	−18 ± 3.14
Wormwood nanoemulsion	160.8 ± 0.038	0.6	−7 ± 3.16

### Larvicidal activity of essential oils, binary combinations, and nanoemulsions against *Culex pipiens* larvae

The larvicidal activity of dill, lime, and wormwood essential oils, their binary combinations, and nanoemulsions against 3rd-instar *Culex pipiens* larvae is presented in Tables 6, 7 and 8. All tested formulations exhibited time and dose-dependent effects. The solvent control produced no mortality, whereas the reference acaricide, Deltamethrin, caused 100% mortality 3 h post-application.

**Table 6:** Larvicidal activity of dill, lime and wormwood essential oil against *Culex pipiens* larvae.

Essential oil/time	Concentration (ppm)							df	*P*-value
	4000	2000	1000	500	250	Control	Deltamethrin		
**Dill**									
30 min	100 ± 0.0^e^	100 ± 0.0^e^	86 ± 6.0^de^	68 ± 3.74^c^	48 ± 3.74^b^	0.0 ± 0.0^a^	72 ± 4.9^cd^	(6,28)	< 0.001
1 h	-	-	100 ± 0.0^c^	100 ± 0.0^c^	74 ± 2.44^b^	0.0 ± 0.0^a^	96 ± 2.45^c^	(4,20	< 0.001
3 h	-	-	-	-	100 ± 0.0	0.0 ± 0.0	100 ± 0.0	-	-
**Lime**									
30 min	100 ± 0.0^e^	100 ± 0.0^e^	92 ± 4.89^e^	56 ± 4.0^c^	32 ± 6.74^b^	0.0 ± 0.0^a^	72 ± 4.9^d^	(6,28)	< 0.001
1 h	-	-	100 ± 0.0^d^	64 ± 3.74^c^	38 ± 6.0^b^	0.0 ± 0.0^a^	96 ± 2.45^d^	(4,20)	< 0.001
3 h	-	-	-	66 ± 6.78^c^	38 ± 3.74^b^	0.0 ± 0.0^a^	100 ± 0.0^d^	(3,16)	< 0.001
6 h	-	-	-	66 ± 6.78^c^	38 ± 3.74^b^	0.0 ± 0.0^a^	-	(2,12)	< 0.001
24 h	-	-	-	72 ± 8.0^c^	44 ± 5.09^b^	0.0 ± 0.0^a^	-	(2,12)	< 0.001
48 h	-	-	-	82 ± 4.89^c^	44 ± 5.09^b^	0.0 ± 0.0^a^	-	(2,12)	< 0.001
**Wormwood**									
30 min	100 ± 0.0^d^	64 ± 6.0^c^	40 ± 7.45^b^	6 ± 4.0^a^	4 ± 4.0^a^	0.0 ± 0.0^a^	72 ± 4.9^c^	(6,28)	< 0.001
1 h	-	80 ± 7.07^bc^	62 ± 3.74^b^	6 ± 4.0^a^	12 ± 5.83^a^	0.0 ± 0.0^a^	96 ± 2.45^c^	(5,24)	< 0.001
3 h	-	92 ± 3.94^c^	74 ± 2.44^b^	8 ± 3.74^a^	20 ± 8.74^a^	0.0 ± 0.0^a^	100 ± 0.0 ^c^	(5,24)	< 0.001
6 h	-	100 ± 0.0^c^	74 ± 2.44^b^	14 ± 9.27^a^	20 ± 8.74^a^	0.0 ± 0.0^a^	-	(4,20)	< 0.001
24 h	-	-	74 ± 2.44^c^	50 ± 6.32^b^	42 ± 8.6^b^	0.0 ± 0.0^a^	-	(3,16)	< 0.001
48 h	-	-	74 ± 2.44^c^	62 ± 5.83^bc^	52 ± 6.63^b^	0.0 ± 0.0^a^	-	(3,16)	< 0.001

Data shown as mean ± SE (*n* = 5 replicates, 10 larvae/replicate). Means with different letters in the same row are significantly different (*p* < 0.001, Tukey’s test).

**Table 7:** Larvicidal activity of essential oil combinations against *Culex pipiens* larvae.

Oil combination/time	Concentration (ppm)							df	*P*-value
	4000	2000	1000	500	250	Control	Deltamethrin		
**Dill +lime**									
30 min	100 ± 0.0^d^	84 ± 5.09^c^	0.0 ± 0.0^a^	0.0 ± 0.0^a^	0.0 ± 0.0^a^	0.0 ± 0.0^a^	72 ± 4.9^b^	(6,28)	< 0.001
1 h	-	100 ± 0.0^c^	32 ± 7.34^b^	4 ± 2.44^a^	0.0 ± 0.0^a^	0.0 ± 0.0^a^	96 ± 2.45^c^	(5,24)	< 0.001
3 h	-	-	48 ± 10.19^c^	22 ± 3.74^b^	2.0 ± 2.0^ab^	0.0 ± 0.0^a^	100 ± 0.0^d^	(4,20)	< 0.001
6 h	-	-	90 ± 4.47^c^	38 ± 7.34^b^	12 ± 5.83^a^	0.0 ± 0.0^a^	-	(2,16)	< 0.001
24 h	-	-	100 ± 0.0^d^	72 ± 7.34^c^	20 ± 4.47^b^	0.0 ± 0.0^a^	-	(2,16)	< 0.001
48 h	-	-	-	72 ± 7.34^c^	40 ± 5.47^b^	0.0 ± 0.0^a^	-	(2,12)	< 0.001
**Dill+wormwood**									
30 min	94 ± 4.0^d^	52 ± 7.34^c^	24 ± 6.78^b^	4.0 ± 4.0^a^	0.0 ± 0.0^a^	0.0 ± 0.0^a^	72 ± 4.9^c^	(6,28)	< 0.001
1 h	100 ± 0.0^c^	84 ± 5.09^c^	56 ± 9.27^b^	0.0 ± 0.0^a^	0.0 ± 0.0^a^	0.0 ± 0.0^a^	96 ± 2.^45c^	(6,28)	< 0.001
3 h	-	100 ± 0.0^c^	82 ± 8.60^c^	32 ± 7.34^b^	4.0 ± 2.44^a^	0.0 ± 0.0^a^	100 ± 0.0^c^	(5,24)	< 0.001
6 h	-	-	90 ± 3.16^c^	42 ± 10.19^b^	4.0 ± 2.44^a^	0.0 ± 0.0^a^	-	(3,16)	< 0.001
24 h	-	-	96 ± 2.44^c^	56 ± 8.12^b^	8 ± 5.8^a^	0.0 ± 0.0^a^	-	(3,16)	< 0.001
48 h	-	-	100 ± 0.0^d^	82 ± 4.89^c^	40 ± 5.47^b^	0.0 ± 0.0^a^	-	(3,16)	< 0.001
**Lime+wormwood**									
30 min	64 ± 6.78^c^	36 ± 8.12^b^	0.0 ± 0.0^a^	0.0 ± 0.0^a^	0.0 ± 0.0^a^	0.0 ± 0.0^a^	72 ± 4.9^c^	(6,28)	< 0.001
1 h	100 ± 0.0^c^	58 ± 10.2^b^	10 ± 3.16^a^	0.0 ± 0.0^a^	2.0 ± 2.0^a^	0.0 ± 0.0^a^	96 ± 2.45^c^	(6,28)	< 0.001
3 h	-	88 ± 7.34^c^	22 ± 4.89^b^	0.0 ± 0.0^a^	4.0 ± 2.44^a^	0.0 ± 0.0^a^	100 ± 0.0^c^	(5,24)	< 0.001
6 h	-	88 ± 7.34^c^	32 ± 6.63^b^	2.0 ± 2.0^a^	10 ± 4.47^a^	0.0 ± 0.0^a^	-	(4,20)	< 0.001
24 h	-	90 ± 5.47^c^	48 ± 9.69^b^	16 ± 5.09^a^	16 ± 9.27^a^	0.0 ± 0.0^a^	-	(4,20)	< 0.001
48 h	-	96 ± 2.44^d^	70 ± 10.0^c^	46 ± 6. 8^bc^	38 ± 5.83^b^	0.0 ± 0.0^a^	-	(4,20)	< 0.001

Data shown as mean ± SE (*n* = 5 replicates, 10 larvae/replicate). Means with different letters in the same row are significantly different (*p* < 0.001, Tukey’s test).

All three EOs caused 100% mortality at 4000 ppm after 30 min. At 250 ppm for the same exposure time, mortality was 48%, 32%, and 4% for dill, lime, and wormwood, respectively. Moreover, dill EO at 250 ppm achieved 100% mortality after 1 h, whereas lime and wormwood EOs produced only 44% and 52% mortality, respectively, after 48 h. Statistical analysis showed that the earliest complete mortality (*P* < 0.05) for dill and lime EOs occurred at 2000 and 4000 ppm after 30 min, whereas for wormwood EO it occurred at 4000 ppm only after the same exposure time (Table 6).

Binary EO combinations of dill + lime, dill + wormwood and lime + wormwood were tested for their larvicidal activity against *C. pipiens*. At 4000 ppm, mortality rates after 30 min were 100%, 94%, and 64% for the dill + lime, dill + wormwood, and lime + wormwood combinations, respectively. Moreover, both the dill + wormwood and lime + wormwood combinations achieved 100% mortality after 1 h. At the lowest concentration of 250 ppm, maximum mortality after 48 h was 40%, 40%, and 38% for the dill + lime, dill + wormwood, and lime + wormwood combinations, respectively. Statistical analysis showed that the earliest complete mortality (*P* < 0.05) for the dill + lime combination occurred at 4000 ppm after 30 min, whereas for the dill + wormwood and lime + wormwood combinations, it occurred at 4000 ppm after 1 h. (Table 7).

**Table 8:** Larvicidal activity of dill, lime and wormwood nanoemulsions against *Culex pipiens* larvae.

Nano-emulsion / time	Concentration (ppm)							Deltamethrin	df	*P*-value
	4000		2000	1000	500	250	Control			
**Dill nanoemulsion**										
30 min	100 ± 0.0^d^		94 ± 2.44^d^	64 ± 7.48^c^	56 ± 2.44^c^	22 ± 7.34^b^	0.0 ± 0.0^a^	72 ± 4.9^c^	(6,28)	< 0.001
1 h	-		100 ± 0.0^c^	100 ± 0.0^c^	90 ± 4.47^c^	54 ± 4.0^b^	0.0 ± 0.0^a^	96 ± 2.45^c^	(5,24)	< 0.001
3 h	-		-	-	100 ± 0.0^c^	58 ± 5.83^b^	0.0 ± 0.0^a^	100 ± 0.0^c^	(3,16)	< 0.001
6 h	-		-	-	-	58 ± 5.83^b^	0.0 ± 0.0^a^	-	(1,8)	< 0.001
24 h	-		-	-	-	100 ± 0.0	0.0 ± 0.0	-	-	-
48 h	-		-	-	-	-	0.0 ± 0.0	-	-	-
**Lime nanoemulsion**										
30 min	100 ± 0.0^d^		72 ± 3.74^c^	56 ± 6.78^c^	38 ± 3.74^b^	26 ± 2.44^b^	0.0 ± 0.0^a^	72 ± 4.9^c^	(6,28)	< 0.001
1 h	-		100 ± 0.0^d^	80 ± 5.47^c^	52 ± 4.89^b^	46 ± 4.0^b^	0.0 ± 0.0^a^	96 ± 2.45^d^	(5,24)	< 0.001
3 h	-		-	86 ± 5.09^c^	62 ± 8.0^b^	48 ± 4.89^b^	0.0 ± 0.0^a^	100 ± 0.0^c^	(4,20)	< 0.001
6 h	-		-	94 ± 6.0^c^	66 ± 8.71^b^	50 ± 5.47^b^	0.0 ± 0.0^a^	-	(3,16)	< 0.001
24 h	-		-	100 ± 0.0^c^	74 ± 12.0^bc^	58 ± 8.60^b^	0.0 ± 0.0^a^	-	(3,16)	< 0.001
48 h	-		-	-	78 ± 13.56^b^	58 ± 8.6^b^	0.0 ± 0.0^a^	-	(2,12)	< 0.001
**Wormwood nanoemulsion**										
30 min	76 ± 2.44^d^		54 ± 6.0^c^	16 ± 2.44^b^	0.0 ± 0.0^a^	0.0 ± 0.0^a^	0.0 ± 0.0^a^	72 ± 4.9^d^	(6,28)	< 0.001
1 h	92 ± 5.3^d^		68 ± 11.57^cd^	46 ± 1029^bc^	20 ± 5.47^ab^	2 ± 2.0^a^	0.0 ± 0.0^a^	96 ± 2.45^d^	(6,28)	< 0.001
3 h	100 ± 0.0^c^		88 ± 8.0^c^	52 ± 9.69^b^	20 ± 5.47^a^	2 ± 2.0^a^	0.0 ± 0.0^a^	100 ± 0.0^c^	(6,28)	< 0.001
6 h	-		100 ± 0.0^d^	74 ± 6.78^c^	32 ± 4.8^b^	4 ± 4.0^a^	0.0 ± 0.0^a^	-	(4,20)	< 0.001
24 h	-		-	94 ± 4.0^c^	52 ± 5.83^b^	32 ± 8.60^b^	0.0 ± 0.0^a^	-	(3,16)	< 0.001
48 h	-		-	100 ± 0.0^c^	62 ± 5.83^b^	42 ± 11.1^b^	0.0 ± 0.0a	-	(3,16)	< 0.001

Data shown as mean ± SE (*n* = 5 replicates, 10 larvae/replicate). Means with different letters in the same row are significantly different (*p* < 0.001, Tukey’s test).

For NEs, at 4000 ppm, dill and lime NEs caused 100% mortality after 30 min, whereas wormwood NE required 3 h to reach 100% mortality. At 250 ppm, NEs were more potent than individual EOs and EO combinations. Dill and lime NE caused 100% and 58% mortality after 24 h, respectively, while wormwood NE caused 42% mortality after 48 h. Statistical analysis showed that the earliest complete mortality (*P* < 0.05) at 4000 ppm occurred after 30 min for dill and lime NE, and after 3 h for wormwood NE (Table 8).

Three-way ANOVA revealed significant main effects and interactions for concentration, exposure time, and essential oil formulation on mortality of *C. pipiens* larvae (Table 9).

**Table 9:** Three-way ANOVA of main effects and interactions for mortality in *Culex pipiens* larvae exposed to different essential oil formulations.

Factor	df	F	Sig.
Concentration	5	1752.908	< 0.001
Time	5	278.866	< 0.001
Oil formulation	8	245.858	< 0.001
Concentration x time	22	31.786	< 0.001
Concentration x oil formulation	40	33.487	< 0.001
Time x oil formulation	36	6.181	< 0.001
Concentration x time x oil formulation	107	2.853	< 0.001

While nanoemulsions showed similar efficacy to crude EOs at 4000 ppm, they were significantly more potent at 250 ppm. This suggests the primary advantages of NEs include both enhanced bioavailability at low concentrations and improved solubility and physical stability. Furthermore, mortality occurred more rapidly with individual EOs than with EO combinations. The synergistic factor (SF) for all EO combinations was < 1, indicating antagonism rather than synergism (Table 10).

**Table 10:** The synergistic factors of essential oils (dill, lime, and wormwood) against *Culex pipiens* larvae.

Oil combination	SF (dill)	SF (lime)	SF (wormwood)
Dill + lime	0.191	0.256	-
Dill + wormwood	0.170	-	0.82
Lime + wormwood	-	0.132	0.475

(SF): synergistic factor. (SF > 1): synergism; (SF < 1): antagonism.

#### Toxicity of essential oils, combinations, and nanoemulsions

**Fig. 4:**
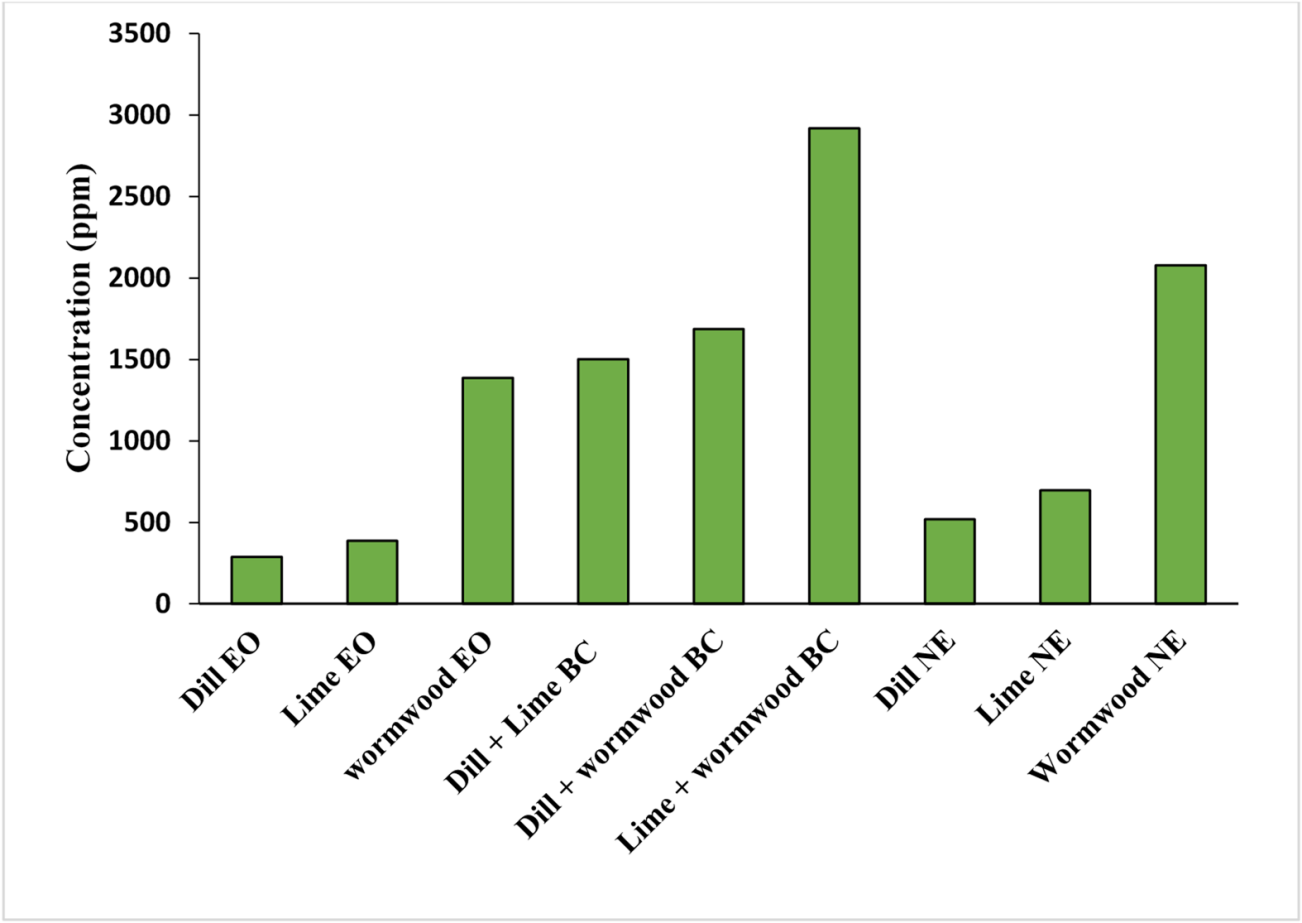
LC_50_ after 30 min of exposure to essential oils, their combinations, and nanoemulsions against *Culex pipiens* larvae. *EO* essential oil; *BC* binary combination; *NE* nanoemulsion.

Based on LC_50_ values, individual EOs exhibited the strongest larvicidal activity, followed by NEs and then EO combinations. For both crude EOs and NEs, the order of efficacy was dill > lime > wormwood. Among EO combinations, those containing dill were most effective (Fig. 4). The larvicidal activity of the tested EOs and their NEs against *C. pipiens* larvae varied. Dill EO exhibited the highest potency with LC_50_ = 286.9 ppm. At 30 min, no significant differences in toxicity were observed between pure EOs and corresponding NEs, as indicated by overlapping confidence interval (CI): dill EO 226.85–341.52 ppm vs. dill NE 290.48–765.12 ppm; lime EO 338.68–432.79 ppm vs. lime NE 279.66–1275 ppm; and wormwood EO 982.43–2092.88 ppm vs. wormwood NE 1827–2372.48 ppm (Table 11). Furthermore, all binary combinations were significantly less toxic than individual pure EOs, as indicated by non-overlapping CI. For example, the dill + lime combination (LC_50_ = 1502.0 ppm; CI: 1403.99–1605.45) had a significantly higher LC_50_ than dill EO alone (LC_50_ = 286.9 ppm; CI: 226.85–341.52) (Table 11).

**Table 11:** Lethal concentration of 50% of *Culex pipiens* larvae (LC_50_) treated with essential oils, their binary combinations, and nanoemulsions after 30 min.

Treatment	LC_50_ (ppm)	Confidence interval (CI) (ppm)	Slope ± SE
Dill essential oil	286.9	226.85 -341.52	2.29 ± 0.265
Lime essential oil	385.98	338.68–432.79	3.2 ± 0.30
Wormwood essential oil	1387.25	982.43- 2092.88	2.69 ± 0.23
Dill + lime combination	1502.0	1403.99–1605.45	7.90 ± 0.762
Dill + wormwood combination	1687.55	1523.56–1873.95	3.46 ± 0.28
Lime + wormwood combination	2918.1	2592–3363	3.25 ± 0.37
Dill nanoemulsion	518.96	290.48 -765.12	2.41 ± 0.20
Lime nanoemulsion	696.7	279.66–1275	1.85 ± 0.162
Wormwood nanoemulsion	2078.7	1827 -2372.48	2.80 ± 0.33

### Effect of dill, lime, and wormwood essential oils and their nanoemulsions on the glutathione S-transferase activity in *Culex pipiens* larvae

Changes in GST activity of *C. pipiens* larvae following exposure to dill, lime, and wormwood EO and their corresponding NEs are presented in Tables 12 and 13. For the EOs, all tested oils reduced GST activity at zero time, 30 min, and 1 h compared with the control (Table 12). With dill EO, GST activity decreased by 28%, 16.2%, and 25.5% at zero time, 30 min, and 1 h, respectively. For lime EO, GST activity was significantly reduced by 47.4% at zero time (*P* < 0.003). After 30 min, activity recovered from 52.6% to 90.5% of the control level, although it remained lower than the control level. By 1 h, GST activity declined again to 56.7% of the control value. Wormwood EO reduced GST activity by 13.6% at zero time relative to the control. After 30 min, enzyme activity nearly returned to the control level. However, after 1 h, GST activity significantly decreased by 53.8% compared with the control (*P* < 0.001). The decline of enzyme activity in larvae exposed to dill EO over time was not statistically significant (*P* > 0.05). However, both lime and wormwood oils showed significant time-dependent differences in enzyme activity (*P* < 0.01). The lowest enzyme activity was recorded at 0 and 1 h for lime oil, and at 1 h for wormwood oil (Table 12).

**Table 12:** Effect of essential oils on the glutathione S-transferase activity for *Culex pipiens* third instar larvae.

Essential oil (EO)	Sp. act. (µmol/min/mg protein) zero time	Sp. act. (µmol/min/mg protein) after 30 min	Sp. act. (µmol/min/mg protein) after 1 h	F _(2,6)_	*P* value
Control (Tween 80)	3.207 ^a^ (100%)	3.915^a^ (100%)	3.247^a^ (100%)	5.612	0.042
Dill	2.3105^a^ (72%)	3.280^a^ (83.8%)	2.417^a^ (74.5%)	1.095	0.393
Lime	1.68^a^ (52.6%)	3.543^b^ (90.5%)	1.841^a^ (56.7%)	17.905	0.003
Wormwood	2.7716 ^b^ (86.4%)	3.9404 ^c^ (100%)	1.503 ^a^ (46.2%)	68.858	< 0.001

Means with different letters in the same row are significantly different (*p* < 0.05, Tukey’s test).

**Table 13:** Effect of nanoemulsions on the glutathione S-transferase activity for *Culex pipiens* third instar larvae.

Nano-emulsion (NE)	Sp. act. (µmol/min/mg protein) zero time	Sp. act. (µmol/min/mg protein) after 30 min	Sp. act. (µmol/min/mg protein) after 1 h	F _(2,6)_	*P* value
Control (water)	1.983^ab^ (100%)	2.744^b^ (100%)	1.061^a^ (100%)	5.958	0.038
Dill	3.347^a^ (168.8%)	3.061^a^ (111.5%)	2.290^a^ (70.77%)	1.520	0.292
Lime	3.259^b^ (164.3%)	2.623^ab^ (96%)	1.455^a^ (137%)	9.273	0.015
Wormwood	4.302^b^ (217%)	2.956^ab^ (107.7%)	1.4674^a^ (138%)	9.3185	0.014

Means with different letters in the same row are significantly different (*p* < 0.05, Tukey’s test).

In contrast to EO, most NE treatments enhanced GST activity, except dill NE at 1 h and lime NE at 30 min, which reduced activity. In larvae treated with dill NE, GST activity increased relative to control by 68.8% at zero time and by 11.5% after 30 min. After 1 h, enzyme activity declined 29.33% in comparison to the control. Lime NE exhibited a different temporal pattern, with GST activity increasing by 64.3% at zero time, decreasing by 4% after 30 min, and then rising significantly by 37% after 1 h. Wormwood NE produced a response distinct from that of wormwood essential oil, as GST activity increased to 217% of the control at zero time. The activity then declined to 107.7% and 138% after 30 min and 1 h, respectively. No significant change in enzyme activity was observed over time in larvae treated with dill NE (*P* > 0.05). In contrast, larvae exposed to lime and wormwood NEs differed significantly over time (*P* < 0.05). For both lime and wormwood NEs, enzyme activity was significantly lower at 30 min and 1 h than at zero time (Table 13).

Two-way ANOVA showed that exposure time, essential oil formulation, and their interaction had significant effects on *C. pipiens* larval GST activity (Table 14).

**Table 14:** Two-way ANOVA of main effects and interactions for the glutathione S-transferase activity in *Culex pipiens* larvae exposed to LC_50_ concentrations of different essential oil formulations.

Factor	df	F	Sig.
Time	2	30.996	< 0.001
Oil formulation	7	4.953	< 0.001
Time x oil formulation	14	3.477	0.001

## Discussion

Mosquito-borne diseases pose a global threat, and increasing resistance to chemical insecticides has intensified interest in developing effective and eco-friendly plant-based products for mosquito control^55^. Mosquito control strategies should primarily target the larval stage, as larvicidal measures are most effective in reducing overall mosquito populations^27,56^. This study tested dill, lime, and wormwood EOs, their binary combinations, and nanoemulsion formulations against *C. pipiens* third-instar larvae. Essential oils are complex mixtures of major and minor aromatic compounds, mainly classified into terpenes, terpene derivatives, hydrocarbons, and other volatile compounds^57,58^. As EOs are predominantly composed of monoterpenes and sesquiterpenes, they show diverse insecticidal properties, including antifeedant, repellent, and toxic effects against a wide range of insects^59,60^.

GC-MS analysis identified the chemical composition of each EO. Dill EO identified Apiol (25.46%), Carvone (24.13%), and D-Limonene (23.47%) as major components, aligning partly with Abdel Karim et al.^61^ who identified apiol (16.16%), D-carvone (37.80%), and D-limonene (18.13%) as the main active constituents. Lime EO main components were D-Limonene (24.16%), α-Terpineol (13.12%), and β-Pinene (11.98%), with Limonene dominance matching with Razzaghi-Abyaneh et al.^62^ and Mohammed et al.^63^. In wormwood EO, the main compounds were davanone (33.8%), camphor (25.67%), and chamazulene (7.47%). These components were nearly similar to Aati et al.^64^, where the major constituents were *cis*-davanone (34.7%), camphor (16.2%), and chamazulene (8.2%). Although the essential oils shared a comparable chemical profile, notable variations were observed in the proportions of their major constituents. These differences in previously reported EO compositions may be attributed to several factors, including the plant geographical origin, the extraction method used, the harvesting time, and whether aerial or floral parts were utilized for oil extraction^65,66^.

In this study, *C. pipiens* larvae were highly susceptible to dill, lime, and wormwood EOs, with 100% mortality after 30 min at 4000 ppm. These EOs have been tested against various mosquito species, but few studies have focused on *C. pipiens*. Dill EO had the highest larvicidal activity (LC_50_: 286.9 ppm). It also showed effectiveness against *Anopheles stephensi* (LC_50_: 38.8 ppm) and *Aedes albopictus* (90% mortality at 0.1 mg/ml)^13^. Additionally, it showed larvicidal and pupicidal activity against *Aedes aegypti* (LC_50_: 0.3% and 2.9% respectively)^14^. The acetone extract of dill was active against *C. pipiens* larvae (LC_50_: 0.071 g/L after 72 h)^68^. Similar to the potent larvicidal activity of dill EO, lime EO showed strong larvicidal activity against *C. pipiens* (LC_50_: 385.98 ppm). These results match Sarma et al.^17^ findings, where *C. aurantifolia* EO showed larvicidal activity against *Aedes aegypti* (LC_50_: 128.81 ppm at 24 h). Additionally, Islam & Akbar^69^ reported the larvicidal effect of lime extract against *Aedes aegypti*, and Soonwera^15^ documented larvicidal activity of lime EO against *Culex quinquefasciatus* (Say). The main constituents of the EO usually influence its effectiveness^70^. Therefore, the larvicidal effect of lime against *C. pipiens* larvae may be attributed to its major component, limonoids, which can penetrate nerve tissue and impair neural function, potentially inducing seizures in the larvae. This disruption of the nervous system leads to sudden hyperactivity and ultimately larval death^71^.

Although dill and lime EOs showed strong larvicidal activity against *C. pipiens* larvae, wormwood EO was comparatively less effective (LC_50_:1387.25 ppm). Various wormwood extracts have been evaluated against other mosquito species, but not specifically against *C. pipiens*. For instance, acetone, methanol, and chloroform wormwood leaf extracts were tested against *A. aegypti*^21^. Sofi et al.^72^ found ethanolic extract was more effective than methanolic extract against *A. aegypti*, and wormwood extracts also showed insecticidal activity against *Chrysomya albiceps*^73^. Although the essential oils were effective against *C. pipiens* larvae, studying their mode of action offers valuable insights into the mechanisms underlying their biological activity. Differences in how *C. pipiens* larvae responded to the EOs might be due to factors like uptake rates, chorion penetration, detoxification, or the toxicant reaching its target site^74^. EOs kill larvae through multiple ways: targeting digestive/neurological enzymes and damaging the integument^75^. The essential oils in this study are primarily composed of monoterpenoids, which act on insects through multiple routes, including the respiratory system (fumigant effect), cuticle (contact effect), and digestive system (ingestion effect), leading to their insecticidal effects^76^. According to Cruz-Castillo et al.^77^, oxygenated monoterpenes such as linalool, estragole, and citronellal are major bioactive ingredients in many essential oils and have demonstrated promising larvicidal action, particularly against *Aedes aegypti*. These substances frequently affect the nervous system or interfere with metabolic pathways in mosquito larvae, resulting in death. Their lipophilicity enables them to permeate larval cuticles and damage interior tissues.

After assessing the larvicidal activity of individual EOs against *C. pipiens* larvae, their binary combinations were evaluated to enhance EO potency. The primary purpose of using synergistic combinations is to use lower concentrations of the tested materials, which reduces costs and increases effectiveness against the target organisms. Additionally, the higher chemical complexity of mixtures lowers the likelihood of resistance development^78^. Although EO combinations are generally believed to improve larvicidal activity through synergy, the current results showed that the three EO combinations exhibited antagonistic effects against *C. pipiens* larvae, with higher LC_50_ values than those of the individual EOs and synergistic factors less than one. The (dill + lime) combination had the highest larvicidal efficacy, with an LC_50_ of 1502 ppm, followed by (dill + wormwood) at 1687.5 ppm, and then (lime + wormwood) at 2918.1 ppm. The observed antagonism may be attributed to compounds within the oils sharing a similar mode of action or to the influence of minor constituents present in the essential oils. In addition, antagonistic effects in certain combinations may result from one compound altering the behavior of another, thereby affecting its absorption, distribution, metabolism, or excretion, as previously proposed by researchers in the context of drug–molecule interactions^79^. Similar antagonistic effects were seen in other studies, like combinations of (*Allium sativum* + *Mentha piperita*) and (*Ocimum sanctum* + *Mentha piperita*) against *C. quinquefasciatus* larvae^30^. Limonene and β-pinene combinations showed antagonism too, according to Andrade-Ochoa et al.^29^. Sarma et al.^17^ observed antagonism between carvone and sulfur compounds. This indicates that, although the oils demonstrated strong efficacy when tested individually, their activity may be reduced when combined with other oils. As reported by Mahanta & Khaniko^30^, mixtures can exhibit synergistic effects at an optimal volumetric ratio of the constituent essential oils; however, outside this range, the synergism may be diminished or lost.

Although essential oils are regarded as promising natural insecticides, their broader application is limited by several technical challenges, including high volatility and low water solubility^80^. In addition, their rapid degradation and short persistence can substantially reduce their toxic effectiveness^81^. Therefore, EOs can be formulated to reduce the volatility of their active components and maintain their biological activity^33^. In this context, nanoemulsion formulations enhance oil solubility in water and improve overall stability^82^.

Physical characterization confirmed the spherical shape and nanoscale size distribution of the NEs. TEM showed droplet sizes of 40–160 nm, while DLS gave mean hydrodynamic diameters of 164.2 nm (dill), 211.6 nm (lime), and 160.8 nm (wormwood). The polydispersity index (PDI) values (0.2–0.6) indicated good homogeneity and stability. The negative zeta potential values confirmed high colloidal stability and favorable surface charge of the NEs. The small droplet sizes provide a high surface area, facilitating penetration of the nanoemulsions into larval cuticle.

Several nanoemulsions have demonstrated larvicidal activity, including those formulated from basil, cumin^83^, *Cymbopogon commutatus*^38^, and rosemary^84^. In the present study, dill, lime, and wormwood NEs were evaluated against *C. pipiens* larvae. Dill and lime NEs exhibited greater efficacy than the wormwood NE. The enhanced activity of dill NE may be attributed to its particle size and low PDI of 0.2.

In the present study, dill NE exhibited notable larvicidal activity, with an LC_50_ value of 518.95 ppm. To the best of our knowledge, dill NE has not previously been evaluated against *C. pipiens* larvae, although Osanloo et al.^67^ reported its effectiveness against *Anopheles stephensi* larvae. Likewise, lime NE showed considerable potency, with an LC_50_ of 696.77 ppm. However, only a few studies have examined lime NE as an insecticidal agent. A study by Li et al.^85^ evaluated its effect on *Aedes albopictus* larvae and reported 100% mortality at a concentration of 75 ppm. In addition, lime NE demonstrated promising antibacterial activity in a study by Liew et al.^86^ and it has also been reported to possess antifungal properties^87^.

In the present study, *C. pipiens* larvae were less sensitive to wormwood NE than to its corresponding EO, as well as dill and lime NE, with LC_50_ value of 2078.7 ppm. These results agree with those reported by Li et al.^85^ who found that green tea NE showed lower activity than its respective essential oil. The reduced immediate toxicity of NEs compared with crude essential oils may be explained by a controlled-release effect or by changes in the physicochemical properties of the active constituents, such as volatility and lipophilicity, during the emulsification process. Previous studies have documented lower toxicity of NEs compared with crude essential oils. For instance, *Psidium cattleyanum* EO was reported to be more toxic than its inclusion complex against *Aedes aegypti* larvae^88^,  in addition, *Carlina acaulis* NE showed lower activity than its corresponding EO against *Lobesia botrana* larvae (Lepidoptera: Tortricidae)^89^.

In the current study, NEs did not exhibit a statistically superior larvicidal effect compared with their corresponding pure essential oils. The confidence interval values of the pure oils and their respective NEs indicated no significant increase in toxicity during 30 min exposure period. This lack of improved efficacy may be attributed to the short exposure period, which may not have been sufficient for the controlled-release mechanism of the NEs to reach their full potential.

While EOs and their NEs show strong larvicidal potential, assessing their effects on detoxification enzymes, such as glutathione S-transferase (GST), is essential for understanding the biochemical basis of larval mortality. GST enzymes are critical for insect survival during exposure to toxins^90^, and changes in their activity serve as biomarkers of physiological responses to natural and synthetic insecticides. In the present study, GST activity in *C. pipiens* larvae was assessed after exposure to the LC_50_ of dill, lime, and wormwood EOs and their NEs. All EOs reduced GST activity relative to controls at 0 h, 30 min, and 1 h post-treatment. Conversely, NEs generally increased GST activity, except dill NE at 1 h and lime NE at 30 min, which caused reductions. The lower LC_50_ values of EOs relative to NEs reflect differences in detoxification impairment. EOs inhibit GST^91^, blocking biotransformation of toxic metabolites and causing rapid systemic failure. NEs instead induce GST activity, indicating larvae initiate a compensatory detoxification response that ultimately fails, indicating a different kinetic mode of action.

Our findings agree with Al-Nazawi et al.^92^, who reported reduced GST activity in *C. pipiens* larvae following treatment with clove, marjoram, and cinnamon EOs. Similarly, Subahar et al.^93^ demonstrated that eugenol and piperine decreased GST activity in *Aedes aegypti*, suggesting that these volatile compounds may act as direct inhibitors of the enzyme or deplete the available GST pool during initial detoxification attempts. The bioactive constituents of EOs, particularly monoterpenes, exhibit high binding affinity for the active sites of GST enzymes. By competitively occupying these sites, these molecules hinder the conjugation of xenobiotics with glutathione (GSH). This enzymatic blockage disrupts the detoxification pathway, resulting in lethal accumulation of toxic secondary metabolites within larval tissues^94^.

NEs generally increased GST activity across exposure times, which is consistent with previous reports for *Pimpinella anisum* NE^95^, jasmine and peppermint NEs in *C. pipiens* larvae^96^, and sandalwood NE in *C. pipiens* and *Aedes aegypti* larvae^97^. This upregulation likely reflects a compensatory detoxification response. The high surface-area-to-volume ratio and enhanced cuticular penetration of NEs may enable sustained, sublethal delivery of active compounds, prompting larvae to overexpress GST enzymes as a metabolic challenge response^98^. The reduced GST activity observed with dill NE at 1 h and lime NE at 30 min may indicate a toxicological tipping point, where intracellular concentrations of active compounds exceeded the larvae’s capacity for enzyme synthesis, resulting in enzymatic failure and mortality.

The rapid larvicidal activity observed in this study, particularly within the first 30 min after treatment, suggests that the EOs exert a fast toxic effect on mosquito larvae. This rapid action may be linked to disruption of detoxification mechanisms, as evidenced by the reduction in GST activity. To the best of our knowledge, this is the first report demonstrating that these EOs initiate larval mortality within 30 min of exposure. Although the LC_50_ values were higher than those reported in some previous studies, the time required to induce mortality was considerably shorter.

## Conclusion

This study evaluated the larvicidal activity of dill, lime, and wormwood EOs, their binary combinations, and NE formulations against *C. pipiens* larvae. Based on LC_50_ values, dill EO exhibited the highest potency (LC_50_ = 286.9 ppm), while the NEs displayed non-significant enhanced toxicity compared with the pure oils. Furthermore, binary combinations resulted in a significant reduction in larvicidal activity, suggesting an antagonistic relationship between the selected oil pairs at the tested ratios. Enzyme assays showed variable effects: dill, lime, and wormwood EOs reduced GST activity, whereas their corresponding NEs generally increased enzyme activity at most exposure times. This study provides a foundational understanding of the larvicidal potential of these formulations; however, some limitations should be acknowledged, as the experiments were conducted under controlled laboratory conditions, which do not account for environmental variables like UV radiation and temperature fluctuations that affect stability in the field. Also, the toxicity of the formulations used toward non-target organisms was not assessed, representing a critical area for future research.

## Data Availability

Data will be made available on request from the corresponding author.
